# Factors influencing the psychological independence of retired community-dwelling older adults in Japan

**DOI:** 10.3389/fpsyg.2022.1004645

**Published:** 2022-12-15

**Authors:** Yuko O. Hirano, Ranze Tamagawa, Takako Matsumoto

**Affiliations:** ^1^Institute of Biomedical Sciences, Nagasaki University, Nagasaki, Japan; ^2^Department of Health Sciences, Faculty of Medicine, Nagasaki University, Nagasaki, Japan

**Keywords:** retirement, community participation, life skill, psychological independence, Japan

## Abstract

**Background:**

In an aging society, it is necessary to promote successful aging by helping older adults maintain engagement in social activities, especially after retirement. Focusing on psychological independence is critical for helping community-dwelling older adults maintain their ability to do things independently and build appropriate relationships with neighbors. However, shifting one’s mindset from the workplace to the local community can be difficult, especially for Japanese people who prioritize work over local community activities. Few studies have investigated the factors associated with the psychological independence of community-dwelling older adults, so this study examined the factors influencing the psychological independence of older adults in T town, a typical commuter town in Japan.

**Methods:**

A self-administered, anonymous questionnaire was distributed to 246 neighborhood association members aged 65 and older. *T*-test, Pearson’s correlation coefficient, Cronbach’s alpha, and multiple regression analysis were used to develop a model for predicting psychological independence and its two subconstructs, purposefulness and personal accountability.

**Results:**

The results demonstrated that psychological independence, purposefulness, and personal accountability were significantly predicted by having abilities that are useful to other people (β = 0.399, *p* = 0.001; β = 0.277, *p* = 0.019; and β = 0.394, *p* = 0.001, respectively).

**Conclusion:**

The findings suggest that older adults can maintain psychological independence using their existing characteristics, such as hobbies and life skills that are useful to others in the community.

## Introduction

Population aging has become a global issue. The World Health Organization (WHO) has reported that from 2015 to 2050, the proportion of the world’s population over 60 will nearly double from 12% to 22% (World Health Organization, 2021). Therefore, developing a successfully aging society is a common goal of many nations. Successful aging includes avoiding disease and disability, engagement with life, and high cognitive and physical functioning (Rowe and Kahn, 1997). Promoting successful aging is interwoven with policy (Bowling and Dieppe, 2005); to encourage population-level policies that support successful aging in older adults, people must demonstrate an interest in and awareness of the social determinants of health (Urtamo et al., 2019). Researchers’ interest in successful aging can be observed globally, including in Korea (Choi et al., 2020), the United Kingdom (Bowling, 2009), the United States of America (Anton et al., 2015; Howard and Louvar, 2017), Taiwan (Hsu et al., 2018), and Nepal (Gautam et al., 2007).

This study focuses on Japan, which is a super-aging society. Japan categorizes people who are above 65 years as “early older adults” and above 75 as “late older adults.” Laws, statistics, and social security system of Japan have been adjusted to reflect this categorization. Since 2020, 28.7% of the Japanese population was over 65 years old (Statistics Bureau of Japan, 2020). Many studies about successful aging have been conducted in Japan, often focusing on the social role of older adults. For example, Wakasaki et al. (2006) conducted a qualitative analysis and found that for middle-aged Japanese women, successful aging included “participation,” “health,” “fulfillment,” “preparation,” “reconsideration,” and “creation.” Moreover, they found that successful aging comprises physical and mental health, commitment, and interaction in the community. These concepts are related, as shown by Onishi et al. (2006), who found that engaging in recreational activities in the community was correlated with happiness among older adults. In addition, Kono et al. (2004) found that the frequency of going outdoors, a simple prognostic indicator of physical and psychological health, was correlated not only with the ability to perform daily life activities but also with individuals’ social role in the community. Thus, when considering the successful aging of older adults, one must consider social participation. This perspective is especially applicable to retired Japanese older adults who have dedicated their lives to their work and less to the local community (Hosouchi et al., 2007, 105–133).

After retiring, older adults lose their corporate status and how people around them had previously evaluated them. Instead, they focus on defining themselves within their community, forming mutually beneficial relationships with the people around them. In Japanese, the act of retired individuals embarking on rebuilding interpersonal relationships in the local community is called *“chiiki debut.”* They are making a “debut” in their community (Hosouchi et al., 2007, 18). The healthy life expectancy of retired individuals at this stage is likely 10–20 years. As they are expected to live in their communities during this period, community participation is essential to successful aging.

This study used psychological independence (Suzuki and Sakihara, 2003) as a facet of successful aging for older adults. The term “psychological independence” is not widely used in Japan; there are other similar concepts, such as IKIGAI, which contain the multi-faceted concepts derived by Wakasaki et al. (2006). As challenges of recent gerontological studies in Japan, Suzuki (2013) mentioned that discussion on the subjective awareness of life and value of the individual older adults in community participation should be emphasized over the theoretical controversy such as “disengagement or integration” of aging, which is controversial among the theorists. Such controversy does not necessarily reflect the reality of older adults. Therefore, researchers should obtain more empirical data by analyzing the subjective awareness of the individuals in later life. This is particularly true for analyzing subjective and multi-faceted concepts, such as IKIGAI. Considering such conditions, we aim to adopt Suzuki and Sakihara’s (2003) psychological independence scale.

Psychological independence does not simply refer to the ability to do things independently, it also refers to building and maintaining appropriate relationships with other people (Fukushima, 1997). Suzuki and Sakihara (2003) indicated that the concept consists of two sub-concepts, purposefulness and personal accountability. The former stresses the importance of self-orientation for a relatively high quality of life. In other words, purposefulness is an intrinsic factor that orients a person’s well-being. This factor is particularly important for individuals who used to define themselves based on their career (Sahlgren, 2013), which is common in the study location T-town. Conversely, the latter corresponds to the importance of self-determination in one’s life, which is particularly important for older people (Fujisaki, 1998, 2–10).

Furthermore, the recent phenomenon of the nuclear family patterns in Japan has accelerated the ratio of single older adult households and all households with at least one resident above 65 years; it is projected that this ratio will reach up to 23.2% by 2025 (Cabinet Office, 2019). This fact compels older adults to turn to the community for a mutual support system. However, personal accountability plays a role in creating good relationships with other community members.

It is necessary to identify factors that promote older adults’ psychological independence to develop a suitable social environment for the growing older population. However, the number of relevant empirical studies is limited. Therefore, this study developed a model to predict factors associated with the psychological independence of community-dwelling older adults in Japan.

The subjective health of older adults may influence psychological independence. Parra-Rizo and Sanchís-Soler (2021) pointed out that autonomy indicated a higher level of satisfaction with psychical activities and functional ability, especially among female participants. Their study also indicated that stronger social relationships are correlated with better subjective health (Parra-Rizo and Sanchís-Soler, 2021). Psychological independence is also influenced by social capital, which is associated with being embedded in dense community networks (Cornwell, 2011) and enables older persons to empower themselves to be “health promotors” in the community (Martínez-Maldonado et al., 2019). Thus, as personal accountability is necessary to build strong bonds in a community, becoming empowered as a health promoter may encourage personal accountability in community-dwelling older adults. In addition, Park et al. (2019) indicated that community-dwelling older adults demonstrate higher levels of physical and mental vitality than those in assisting living settings. This vitality is made possible because, in the community, older adults can use their social and communication skills and hobbies to support neighbors and avoid conflicts. These activities stimulate physical and mental activities that promote life satisfaction.

## Materials and methods

### Study setting, participants, and data collection

The study was conducted in T town, a suburb of Nagasaki City. During the 1980s, T town was developed as a commuter town with public transportation to Nagasaki City. Thus, town residents did not have prior relationships with each other. Furthermore, most of the residents are corporate employees who work in the city during the week, leading to limited socialization opportunities with the neighbors. Socialization is also limited on weekends as for Japanese corporate employees, the local community outside the corporation becomes a “hollow shell” (Osawa and Scott, 1992). That is, it does not have significant meaning for them. Therefore, when they began their post-retirement life, many lack interpersonal relationships within the community. This made T town a newly developed community, enabling its selection as the study setting. We aimed to examine how the relationships among older adults in a new community predicted psychological independence.

T-town comprised 425 households and 1,063 people at the end of 2017. The proportion of persons aged over 65 years was 27.0% at the end of 2017 and is expected to exceed 40% by 2025 (T-town, 2018). The participants in this study were sampled from T town’s neighborhood association. The neighborhood association membership rate remained at around 90%, a high level compared to Nagasaki City’s average of 72%. In this study, participants included 246 members of the neighborhood association aged over 65. Although, neighborhood association memberships have become unenforced in Japan, we observed that even the non-members of the neighborhood association could participate in the local festival with the members. Considering this condition, we assumed that holding memberships is not a determinant of community participation for the residents of T-town.

A six-page questionnaire was developed. The head of the neighborhood association distributed the questionnaire to the participants *via* mail.

### Measures

#### Psychological independence

We used a four-point Likert scale (1 = *disagree*, 4 = *agree*) developed by Suzuki and Sakihara (2003), which was developed to measure the psychological independence of Japanese adults. It includes two subscales, with four items in each subscale: purposefulness (“I have hobbies and pleasures,” “I have a goal in my life,” “I have something that I can be absorbed in,” and “I want to do something that benefits other people”) and personal accountability (“I am likely to make a decision and do it by myself,” “I am likely to not to be influenced by the conditions or opinions of other people,” “I am responsible for my opinion and actions,” and “I am confident in my opinion”).

#### Subjective burden in daily life

We used a question derived from Imaya et al. (2013): “In your daily life, to what degree do you feel burdened by problems relating to housework, work, the community, and interpersonal relationships?” Responses were measured on a four-point Likert scale (1 = *not feel at all*, 4 = *feel very much*).

#### Subjective well-being

We used a question from the Short Form Health (SF-8) questionnaire (Fukuhara and Suzukamo, 2005): “Generally speaking, how healthy have you been during the past month?” Responses were measured on a five-point Likert scale (1 = *very bad*, 6 = *very good*).

#### Social capital

Social capital was measured with three questions derived from the Cabinet Office’s Questionnaire Survey on Social Capital (Cabinet Office, 2002). First, the degree of trust in neighbors was measured by asking, “How much do you trust your neighbors?” Responses were measured on a four-point Likert scale (1 = *do not trust at all*, 2 = *trust a little*, 3 = *trust*, 4 = *trust very much*). Second, cooperation with neighbors was measured by asking, “How much do you accept relationships with your neighbors?” using a four-point Likert scale (1 = *not acceptable at all*, 2 = *somewhat not acceptable*, 3 = *somewhat acceptable*, 4 = *acceptable*). Third, mutual support among neighbors was measured by asking, “How do you describe the relationship with your neighbors most? Select one from the below: (i) We seldom have contact unless we are at the community notice board, (ii) We greet each other when we meet at a community event, (iii) We have a chat when we meet, and (iv) We ask for help or exchange opinions in various matters.”

#### Knowledge and skills that are useful to other people

We measured participants’ social skills that can contribute to the community with the following question: “To what degree do you have knowledge, skills, and hobbies that are likely to be useful to other people (e.g., can babysit and look after children; do you know about household appliances, etc.)?” A four-point Likert scale (1 = *not at all*, 2 = *a little*, 3 = *somewhat*, 4 = *many*) was used. In addition, useful knowledge and skills types were reported in response to an open-ended question.

#### Ability to avoid conflict in interpersonal relationships

We measured this with the following question: “When you disagree with other people, to what extent are you aware of making an effort to avoid clashes, such as by making humorous remarks?” This item was extracted from an original scale developed by Kunugimoto and Yamasaki (2010). A four-point Likert scale (1 = *not at all*, 4 = *very much*) was used.

#### Comments

Finally, an open-ended question was included, asking for any comments about the survey.

### Statistical analysis

IBM SPSS Version 22 was used to conduct statistical analysis. First, we conducted a *t*-test on the study’s categorical data (i.e., gender, current employment status). In addition, we used the Pearson’s correlation coefficient to find the correlation between the independent variables (i.e., the degree of subjective burden in daily life, subjective well-being, degree of trust in neighbors, degree of cooperation with neighbors, mutual support with neighbors, having abilities that are useful to other people, and ability to avoid conflicts in interpersonal relationships) and psychological independence. Cronbach’s alpha was used to estimate the reliability of psychological independence. Finally, a multiple regression analysis was conducted to develop a predictive model for psychological independence. The multiple regression analysis used social-demographic characteristics (gender, age, current employment status, length of residency by year, and family structure) as control variables. When selecting the independent variables in multiple regression analysis, we used a variance inflation factor to confirm that there were no problems due to multicollinearity. A probability of <0.05 was considered statistically significant.

### Ethical considerations

The study was conducted after obtaining approval from the Ethical Review Board of Nagasaki University (Registration No: 18071209). Informed written consent was obtained from all participants.

## Results

### Participants

Data from 112 participants were analyzed (response rate: 45.5%). Table 1 shows the participant socio-demographic information.

**Table 1:** Social-demographic characteristics of participants (*N* = 112).

	Item	Average (±SD) or *n* (%)
Age in years	Average (SD)	72.5 (±6.1)
Gender	Male	59 (55.7%)
	Female	47 (44.3%)
Currently employed	Yes	29 (27.6%)
	No	76 (72.4%)
Family structure	Living alone	39 (36.1%)
	Living with others	69 (63.9%)
Length of residency in years	Average (SD)	25.9 (±8.3)
How much do you accept relationship with your neighbors?	Not acceptable at all/somewhat not acceptable	13 (11.6%)
	Somewhat acceptable/acceptable	96 (85.7%)
How much do you trust your neighbors?	Trust / trust very much	75 (66.9%)
	Do not trust at all/trust a little	36 (32.1%)
How do you describe the relationship with your neighbors most?	We seldom have contact unless we are at the community notice board	19 (17.0%)
	We greet to each other when we meet at a community event	57 (50.9%)
	We have a chat when we meet	21 (18.8%)
	We ask for help or exchange opinions in various matters	11 (9.8%)

### Mono-variate analysis of the independent variables

The average scores were as follows: 2.64 (± 0.82) for the degree of subjective burden in daily life, 3.73 (± 0.90) for subjective well-being, 2.81 (± 0.84) for the degree of trust in neighbors, 2.77 (±0.88) for the degree of cooperation with neighbors, 3.11 (±0.61) for mutual support among neighbors, 2.46 (± 0.80) for having abilities that are useful to other people, and 3.14 (± 0.65) for the ability to avoid conflict in interpersonal relationships.

The average scores were as follows: 26.5 (±3.77) for psychological independence (Cronbach’s α = 0.79), 13.2 (±2.46) for purposefulness (Cronbach’s α = 0.74), and 13.3 (±1.96) for personal accountability (Cronbach’s α = 0.73).

### Factors related to psychological independence

#### Socio-demographic characteristics and psychological independence

No significant relationships were observed between socio-demographic characteristics and psychological independence.

#### Correlation between independent variables and psychological independence

A significant correlation was observed between psychological independence and (i) having abilities that are useful to other people (r = 0.450, *p* < 0.001), (ii) subjective well-being (r = 0.318, *p* < 0.001; Table 2).

**Table 2:** Correlations between independent variables and psychological independence.

	1	2	3	4	5	6	7	8	9	10	11
1. Age											
2. Length of residency	0.032										
3. Subjective burden in daily life	0.005	−0.059									
4. Subjective well-being	0.070	0.143	−0.235^*^								
5. Trust in neighbors	0.288^**^	0.040	0.015	0.310^***^							
6. Cooperation with neighbors	0.030	−0.056	−0.037	0.187	0.224^*^						
7. Cognition of mutual support with neighbors	−0.004	−0.109	−0.064	0.085	0.113	0.317^***^					
8. Having abilities that are useful to other people	0.056	−0.101	0.059	0.170	0.361^***^	0.163	0.271^**^				
9. Ability to avoid conflicts in interpersonal relationships	0.091	0.034	0.102	0.192^*^	0.232^*^	0.297^***^	0.108	0.340^***^			
10. Psychological independence	−0.046	0.077	0.028	0.318^***^	0.232^*^	0.215^*^	0.210^*^	0.450^***^	0.314^***^		
11. Purposefulness	−0.139	0.181	0.064	0.218^**^	0.209^*^	0.189	0.183	0.333^***^	0.285^***^	0.887^***^	
12. Accountability	0.034	−0.066	−0.063	0.348^***^	0.180	0.171	0.178	0.442^***^	0.248^*^	0.819^***^	0.461^***^

* *p* < 0.05;

** *p* < 0.01;

*** *p* < 0.001.

#### Factors related to psychological independence (multiple regression analysis)

The results of the multivariate analysis demonstrated that psychological independence and its sub-concepts were significantly correlated with having abilities that are useful to other people (β = 0.399, *p* = 0.001; β = 0.277, *p* = 0.019; β = 0.394, *p* = 0.001), respectively. Subjective well-being was significantly correlated with psychological independence (β = 0.259, *p* = 0.013) and personal accountability (β = 0.314, *p* = 0.003; Table 3).

**Table 3:** Factors relating to psychological independence and its subscales.

Item	Psychological independence		Purposefulness		Personal accountability	
	β	*p-*value	β	*p*-value	β	*p*-value
Age	−0.123	0.235	−0.199	0.064	−0.007	0.947
Gender (male = 1, female = 2)	0.118	0.229	0.167	0.101	0.005	0.958
Current employment (Yes = 1, No = 2)	0.017	0.870	−0.030	0.776	0.077	0.459
Length of residency	0.112	0.249	0.176	0.081	−0.005	0.961
Family structure (Living alone = 1, Living with others = 0)	−0.005	0.957	−0.023	0.826	−0.010	0.923
Subjective burden in daily life	0.020	0.831	0.060	0.545	−0.059	0.541
Subjective well-being	0.259	0.013	0.140	0.190	0.314	0.003
Trust in neighbors	−0.028	0.803	0.050	0.666	−0.094	0.409
Mutual support with neighbors	0.004	0.967	0.009	0.934	−0.008	0.938
Cooperation with neighbors	0.096	0.349	0.120	0.264	0.044	0.673
Having abilities that are useful to other people	0.399	0.001	0.277	0.019	0.394	0.001
Ability to avoid conflicts in interpersonal relationships	0.074	0.492	0.080	0.473	0.062	0.568
*R* ^2^	0.331	0.001	0.273	0.007	0.308	0.002
Adjusted *R*^2^	0.231		0.165		0.205	

#### Free answers about abilities that are useful to other people

The qualitative data obtained through open-ended questions were divided into three categories: “those that make use of knowledge gained at work” (24 cases), “those that make use of a hobby” (30 cases), and “those that make use of life skills” (18 cases).

## Discussion

This study investigated factors associated with the psychological independence of community-dwelling older adults. The findings suggest that psychological independence can be enhanced by promoting subjective well-being and using the respondents’ existing characteristics. Interestingly, having abilities that are useful to other people was moderately associated with cooperation with neighbors, mutual support, conflict-avoidance in interpersonal relationships, and psychological independence. Notably, a multi-regression analysis identified abilities that are useful to other people as the strongest indicator of psychological independence.

The open-ended study question identified three categories of older adult abilities that are valuable to others; namely, knowledge gained at work, hobbies, and life skills. An example of knowledge gained at work is as follows:

*I am happy to help those who are not able to fix their electric appliances. I used to do electric appliance repair before I retired.* (A retired engineer)

He felt comfortable when he found himself contributing to the local community using the skills he obtained while working as an engineer. Similar narratives of life skills are also reported by Yan (2021). For Japanese older adults, volunteer work is more frequently observed among women than among men (Takeda et al., 2022), and it is effective for easing the feeling of isolation (Yan, 2021). Engaging in volunteer work can even restrain the onset of depression in the following 3 years (Tamura et al., 2021). Such voluntary acts oriented him to compassion for others (other-oriented), and provided him with worthwhile, engaging activities (self-oriented). It is understood that the stronger both orientations are, the greater the satisfaction will be with engaging in volunteer activities (Ito, 2019). Notably, this case is compatible with the government’s “lifetime player” campaign (Ministry of Health, Labour and Welfare, 2019) to mobilize the skills and knowledge of retired people in community settings. As mentioned previously, the “lifetime player” campaign could benefit the community and those who seek to utilize their skills and knowledge.

Hobbies are the most frequent answer given by older adults related to assisting others in this study. Some examples of hobbies are, *ken-do*, a traditional Japanese martial arts form, in which they can instruct others. As *ken-do* requires maturity in performing, it is a sport that older adults, among themselves, and younger generations enjoy equally (Asami et al., 1996). Making *tsukemono*, a traditional pickle snack to offer to others, is a frequently observed activity in households with older residents (Nakamura et al., 1989). These activities may boost the psychological independence of older adults by utilizing their hobbies to contribute to the community.

Hobbies are popular among older adults, particularly those aged 65–74 (Toepoel, 2013). It has been shown to enrich the quality of life in community-dwelling older adults (Hirano et al., 2017; Fukase et al., 2018) and even in an evacuation site of natural disasters (Xie et al., 2017). It enhances psychological independence, regardless of physical impairments, such as being near-sighted (Lamoureux et al., 2009). Hobbies provide opportunities for individuals to be absorbed in an activity (Yanagihara and Kobayashi, 2018), which then enhances the subjective well-being of older adults, including those who receive care at home (Hayasaka et al., 2002). These examples demonstrate that having hobbies elevates individuals’ purposefulness. Therefore, they might be suitable for shifting older adults’ focus from a corporate-centered to a more community-centered lifestyle. Having hobbies also helps promote connectedness between individuals. As indicated in Table 2, having hobbies that are useful to other people is associated with trust in neighbors and cognition of mutual support. Interestingly, it is also associated with avoiding conflicts in interpersonal relationships. This finding is supported by a study by Toepoel (2013), which found that hobbies are a predictor of social connectedness, and a study by Arslantaş et al. (2015), which found that a lack of hobbies is a strong predictor of loneliness.

Life skills, such as making other people laugh, as one study respondent offered, may help individuals avoid conflicts and deepen interpersonal relationships. The WHO has stressed the importance of life skills as “abilities that enable individuals to behave in healthy ways, given the desire to do so and given the scope and opportunity to do so” (World Health Organization, 1997). Older adults are required to obtain a broad range of new life skills. *Role recognition skills* involve adjusting to a new environment after retirement, enabling them to be aware of social expectations and demands. *Role execution skills* enable them to discover alternative activities in place of their former occupations. Older adults must develop skills to invigorate and inspire their lives going forward post-retirement. These skills are needed to build psychological independence. They must reconfigure and reset life goals, use environmental resources, and actively process and modify the environment (Oda, 2004). Thus, the ability to promote laughter and camaraderie in the community, as suggested above, could be one effective strategy for maintaining psychological independence.

The results indicate that having abilities that are useful to other people, namely utilizing knowledge gained at work, hobbies, and life skills, is vital for community participation, especially for those who are “debuting” in their neighborhood after retirement. In addition, community participation requires individuals to be accountable for the activities in which they engage. Community participation also corresponds to a sense of purposefulness in life. Therefore, psychological independence, which contains facets of purposefulness and personal accountability, was indicated by having abilities that are useful to other people. This finding that having useful abilities is important is compatible with the results of previous studies that have indicated that community participation is a significant indicator of the quality of life (He et al., 2017; Choi et al., 2020) and successful aging (de Moraes and de Azevedoe Souza, 2005). Thus, the study may contribute to future research about community participation in commuter towns in Japan, many of which face the imminent issues of how to support retired residents.

Currently in Japan, various programs are being implemented to promote community participation (Senuma, 2006). Through these programs, community residents can “try to spontaneously and autonomously organize and expand their own lives, so that they can actively engage in the community participation throughout the lifetime” (Kanau, 2008). Older adults’ quality of life after retirement is enhanced, and the entire community is invigorated. As a measure to promote such community participation, Hosouchi et al. (2007, 105–133) recommended that older retirees become entrepreneurs and start community businesses designed to energize the community, with the residents taking the lead and ensuring that the human, social, and economic qualities are in balance. Going forward, it will become essential to implement these programs throughout Japan. The Japanese government expects older adults to be “lifetime players” by providing the right “place” and “role” for them to perform (Cabinet Office, n.d.). Furthermore, we believe that supporting the formation of a society where older adults can enjoy a high quality of life during retirement by forming interpersonal relationships will become even more critical in the coming years due to Japan’s super-aging society.

Despite the study’s contribution to the field, there are some limitations. First, there may have been a sampling bias. The participants were adults aged 65 and over, referred to us by the president of a neighborhood association. Thus, those who were not members of the association were not included in the survey. Second, the characteristics of T-town may have influenced the results, as participation in the neighborhood association was relatively high compared to other regions. This characteristic may have influenced the study participants’ recognition of social relationships with neighbors. For this reason, the results may not be generalizable to other areas, and further studies are needed to analyze the psychological independence of those who do not belong to a neighborhood association. Third, the academic achievements of older adults, which is an indicator of volunteer engagement (Mitani, 2016), was not included in the questionnaire. Therefore, future studies are suggested to include these indicators.

## Data availability statement

The raw data supporting the conclusions of this article will be made available by the corresponding author on reasonable request.

## Ethics statement

The studies involving human participants were reviewed and approved by Ethics Committee of Graduate School of Biomedical Sciences, Health Science Courses, Nagasaki University (no. 18071209). The patients/participants provided their written informed consent to participate in this study.

## Author contributions

RT and TM were responsible for developing a questionnaire, conducting the survey, and performing data analysis under the supervision of YH. YH completed the basic writing of the manuscript. All authors contributed to the article and approved the submitted version.

## Funding

This study was funded by Management Expenses Grants, Nagasaki University.

## Conflict of interest

The authors declare that the research was conducted without any commercial or financial relationships that could be construed as potential conflicts of interest.

## Publisher’s note

All claims expressed in this article are solely those of the authors and do not necessarily represent those of their affiliated organizations, or those of the publisher, the editors and the reviewers. Any product that may be evaluated in this article, or claim that may be made by its manufacturer, is not guaranteed or endorsed by the publisher.
